# Determination of Methamphetamine by High-Performance Liquid Chromatography in Odor-Adsorbent Material Used for Training Drug-Detection Animals

**DOI:** 10.3390/molecules29051091

**Published:** 2024-02-29

**Authors:** Ning Sun, Jingjing Chao, Xiaochang Liu, Hao Li, Dongshun Jia, Dajun Zhang, Liuwei Xie, Yuanting Zhou, Wenxuan Lang, Yingyi Shui, Qiwen Zhu

**Affiliations:** 1 Police Dog Technical College, Criminal Investigation Police University of China, Shenyang 110048, China; sunningcipuc@126.com (N.S.); cindy304@163.com (J.C.); xieliuwei@cipuc.edu.cn (L.X.); 18621504719@163.com (Y.Z.); 2 Liaoning Provincial Key Laboratory of Behavioral Cognition, Shenyang Medical College, Shenyang 110034, China; liuxiaochang1991@163.com (X.L.); lh1330891@163.com (H.L.); 15907425055@163.com (D.J.); zhangdajun2008@126.com (D.Z.); x728753967@163.com (W.L.); 3 Police Dog Team, Criminal Investigation Corps, Shanghai Public Security Bureau, Shanghai 201799, China; 4 Information Network Security College, People’s Public Security University of China, Beijing 100038, China; xxsyy0531@163.com

**Keywords:** methamphetamine, high-performance liquid chromatography (HPLC), drug-detection animals, adsorbent material, content determination

## Abstract

The objective of the present report was to develop and validate a simple, sensitive, and selective analytical method for the determination of methamphetamine in an odor-adsorbent material (gauze) which was used to improve and standardize the training method used for drug-detection animals. High-performance liquid chromatography (HPLC) was performed using a Spherisorb ODS2 C18 column (200 mm × 4.6 mm, 5 μm), with a mobile phase consisting of a 0.25% methanol/triethylamine aqueous solution (V:V = 20:80), the pH of which was adjusted to 3.1 using glacial acetic acid, at a flow rate of 1.0 mL/min. The column temperature was 25 °C, and the detection of the analytes was performed at a wavelength of 260 nm. Methamphetamine showed good linearity (*R*^2^ = 0.9999) in the range of 4.2~83.2 mg/mL. The stability of the test material was good over 24 h. The precision of the method was good, with an average spiked recovery of 86.2% and an RSD of 2.9%. The methamphetamine content in the gauze sample was determined to be 7.8 ± 2.2 μg/sample. A high-performance liquid chromatography (HPLC) method was optimized and validated for the determination of methamphetamine in adsorbent materials (gauze). Validation data in terms of specificity, linearity, the limit of detection and the limit of quantification, reproducibility, precision, stability, and recovery indicated that the method is suitable for the routine analysis of methamphetamine in adsorbent materials (gauze) and provided a basis for training drug-detection animals.

## 1. Introduction

Methamphetamine (MA) is a euphoric amphetamine-type drug that is abused worldwide. Methamphetamine acts on the central nervous system to release monoamines (dopamine, norepinephrine, and serotonin) [1] which are capable of causing intense excitation of the central nervous system, manifested by symptoms such as agitation, restlessness, and convulsions, which can cause cardiovascular disease, paranoia, psychosis, and even death [2,3].

Illicitly manufactured and sold, methamphetamine is generally made in the form of hydrochloride, i.e., methamphetamine hydrochloride, in clear crystals, known as crystal methamphetamine (CM), which are of the highest purity and are therefore more potent [4]. Methamphetamine hydrochloride is not volatile, but methamphetamine is volatile, and due to its unstable chemical constitution, methamphetamine exists in air as a volatile free base [5]. Methamphetamine is manufactured in clandestine laboratories through a variety of methods (referred to as “cooking”) [6]. Volatile organic compounds (VOCs) from methamphetamine are released into the air during the manufacturing process and after drug-taking [7]. Methamphetamine was reported in indoor air at levels between 0.2 and 7.3 µg/m^3^ in a clandestine laboratory seized by police in the US [8]. Methamphetamine residues can be deposited on the surface of an object and spread further through the skin and mouth; they can also be airborne [9]. Methamphetamine is transferred to structures that are porous on the surface and remains there for a long time [9]. The manufacture and abuse of methamphetamine and the contamination it causes are now major problems affecting public health, the economy, and the environment.

Given the volatile and contaminating nature of methamphetamine, an animal’s sense of smell can be used to its advantage. As trace chemical detectors, animals (e.g., dogs and rodents) rely on their remarkable olfactory abilities to detect specific odors and have been widely used in illicit drug detection [10], explosive searches [11], mine detection [12], search and rescue operations [13], and cadaver detection [14], with increasing interest in their use in forensic medicine and security [15,16]. The application of animals as trace chemical detectors is currently expanding to medical fields such as cancer detection [17] and blood glucose testing [18] due to its non-invasive nature and early detection of disease. Dogs are the most typical representatives. After training, drug-sniffing dogs routinely inspect people and goods at airports, ports, and postal warehouses, searching for a variety of drugs such as cocaine, heroin, methamphetamine, and marijuana [19], and drug-detection dogs have made a great contribution to human society in the interception of drug smuggling.

In the training of odor-detection animals, there are usually three forms of choice for odor training aids: the true material, pseudo-odors, and non-pseudo alternatives. The true material refers to the target substance itself, i.e., methamphetamine in this study; pseudo-odors are the chemical components of the true material which are now commercialized, such as the products developed by Sigma-Aldrich in the U.S.; and non-pseudo alternatives refer to the use of special ways of converting the odor of the true material, including its adsorption, dilution, encapsulation, and extraction. Among these forms of odor training aids, the true material is considered the most effective for training, but due to its safety, transportation, cost, storage, handling, etc., there are problems of difficult access and management. Currently, in police dog training, it is common to use the adsorption/absorption method with pseudo-odors. The adsorption method is to adsorb/absorb the odor of the true material onto/into the secondary material, which is non-initiatory, non-toxic, and non-infectious (e.g., COVID-19) [20].

Common secondary materials include steel, cotton, or a polymer. For example, Dutch police dog trainers have used steel tubes to collect human odors, a method that facilitates cleaning and odor removal but also suffers from the uneven collection of target odors [21]. In some ways, it is the same as the method of training dogs to search for physical evidence, both of which involve a person briefly touching an object and leaving an odor on the object. Natural fibers such as cotton are the most commonly used adsorbent materials. Currently, there are two types of odor-adsorption methods used with cotton: static and dynamic. Static adsorption involves placing cotton in the vicinity of the true material to collect its odor; dynamic adsorption involves pumping the odor to the cotton using a device such as the STU-100 [22]. In police dog training practice, gauze is often used to collect odor statically. Gauze is a sparsely woven cotton fabric with a sparse warp and weft; the main raw material used in its production is cellulose, which is porous and in which odors tend to be well preserved, but its ability to adsorb or release odors depends on the type of material. The U.S. Federal Bureau of Investigation (FBI) uses sterile gauze (Johnson), a mixture of cotton, rayon, and polyester, while the Dutch National Police (DNP) use cotton (King’s Cotton) [21], and most of China uses commercially available sterile gauze. Animals trained with this material quickly learn to respond positively to the true material, which just proves the suitability and efficacy of gauze as a training aid. However, quantitative analysis studies of target odors in adsorbent materials (gauze) during animal training are lacking. There is an urgent need for a simple, sensitive, and selective analytical method for the determination of methamphetamine in an odor-adsorbent material (gauze) for training drug-detection animals.

In order to prevent the widespread distribution and abuse of methamphetamine, various techniques have been developed for the detection of methamphetamine, including liquid chromatography (LC) [23], gas chromatography (GC) [24], capillary electrophoresis [25], immunoassays [26], mass spectrometry (MS), and molecularly imprinted polymer solid-phase extraction [27]. Detection methods for methamphetamine have been changing rapidly in recent years, as recorded in the “Interpol Review of Drug Analysis 2019–2022” [28]; fluorescent nanosensors and the supercritical fluid chromatography–tandem mass spectrometry method were, respectively, used to conduct methamphetamine and methylamphetamine impurity profiling in 2019; in 2020, novel fluorescent nanosensors based on graphene quantum dots in molecularly imprinted polymers were found to be powerful tools for methamphetamine analysis [29]; and in 2022, forensic markers of the 1-phenyl-2-propanone synthesis pathway were investigated to identify precursors to methamphetamine [30].

Overall, GC and LC are the most commonly used methods for the detection of methamphetamine [5]. Al-Dirbashi et al. [31] used high-performance liquid chromatography (HPLC), and Wright, Martyny, and Serrano and Van et al. [5,32,33,34] used GC and LC techniques, respectively. Although GC is highly sensitive, qualitatively accurate, and widely used, LC has the advantages of less demanding sample preparation and independence from sample volatility and polarity as LC does not require derivatization to enhance volatility and is capable of characterizing and quantifying thermally unstable and polar compounds [35]. Therefore, compared to GC, LC may have some advantages in this regard.

The olfactory detection capabilities of animals are comparable to chemical analytical instruments; however, significant challenges remain for the use of odor as evidence in court, and the reliability and accuracy of animal olfactory capabilities must be further rigorously validated. Our aim in this study was to develop and validate a simple, sensitive, and selective analytical method for the detection and quantification of methamphetamine in odor-adsorbent materials that could be used to guide training methods for the detection of odors by animals which, in turn, could be refined and standardized to expedite the process of using odors as evidence in court.

## 2. Results and Discussion

### 2.1. The Optimization of the Analytical Method

During method development, chromatographic conditions were optimized to achieve the best separation of methamphetamine.

#### 2.1.1. Selection of Detection Materials for Odor Adsorption

The sampling and detection of methamphetamine on hard surfaces (e.g., walls, gypsum board/plaster walls, ceilings, plastics, and glass) were reported previously [7,36,37]. In terms of detection materials, methamphetamine is sampled and detected in biological samples, ambient air, and illegal samples or on contaminated surfaces, with a particular predominance of biological samples such as blood, urine, saliva, and hair. Concheiro et al. [38] used a liquid–liquid extraction method to detect seven amphetamine-type drugs in urine, including amphetamine and methamphetamine. Wood et al. [39] detected six amphetamine-type drugs in blood. Strano-Rossi et al. [40] used a saturated carbonate solution with a pH of 10 as the extractant, controlled the temperature at 40 °C, and performed ultrasonic liquid–liquid extraction for 30 min to extract five amphetamine-type drugs from hair. Meng et al. [41] used 1 mol/L of NaOH to release four amphetamine drugs from hair. Bjørk et al. [42] extracted nineteen drugs, including amphetamine, cocaine, and morphine, from blood using a solid-phase extraction pretreatment. Kataoka et al. [43] used acetonitrile (50 mM) ammonium acetate as an eluent with a volume ratio of 15:85 for isocratic elution, and a column was chosen to analyze five amphetamine drugs by LC-MS using cyanotoxins (LC-CN, 4.6 mm × 3.3 cm, 3 μm).

There is research evidence that contact with contaminated clothing and fabrics could be due to exposure to methamphetamine [44]. Al-Dirbashi et al. [31] detected methamphetamine in underwear and trouser samples from drug users using high-performance liquid chromatography–fluorescence (HPLC-FL) or high-performance liquid chromatography–ultraviolet (HPLC-UV) methods. Similarly, Keasey [45] detected methamphetamine residues in clothing samples from drug users by liquid extraction and gas chromatography–mass spectrometry (GC-MS). Martyny et al. [46] used phosphorus and anhydrous ammonia methods to take surface wipe samples of protective clothing worn by drug makers and then quantified methamphetamine extracted from the clothing, which showed that methamphetamine levels ranged from 0.2 to 150 μg/sample. This suggests that airborne methamphetamine can be transferred and contaminate clothing, i.e., methamphetamine can be extracted from clothing and fabrics. Loose and porous cotton material readily absorbs methamphetamine [33]. Experiments by Morrison et al. [44] also demonstrated that compared to polyester with fewer polar sites, cotton with more polar sites has a stronger absorption capacity for polar methamphetamine. In the NIOSH Manual of Analytical Methods (NMAM) handbook published by the National Institute for Occupational Safety and Health (NIOSH), it is stated that gauze is the wipe medium of choice and is better than five-layer synthetic fiber gauze for the detection of methamphetamine [47]. This indicates that gauze is an ideal medium suitable for the detection of methamphetamine, both for surface wipe sampling and for odor adsorption material selection.

#### 2.1.2. Selection of Detection Wavelength

**Figure 1:**
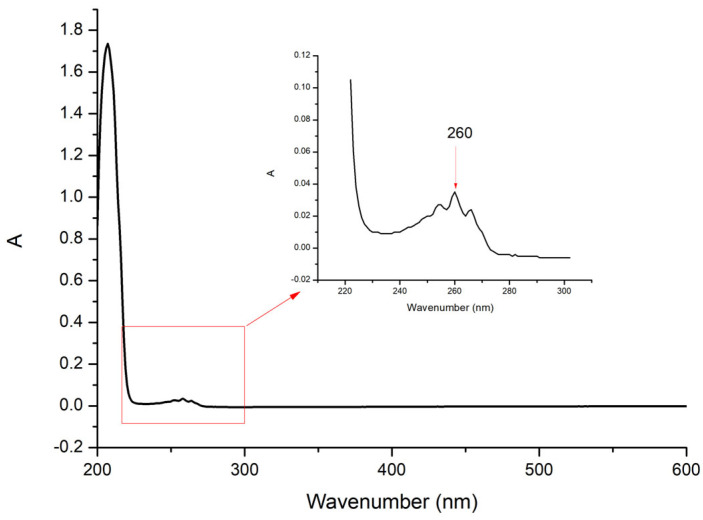
Full-wavelength UV scan of methamphetamine.

The detector in this experiment was an ultraviolet detector (UV). Using methanol as a reference solution, an appropriate amount of methamphetamine was dissolved and diluted with methanol and then scanned in the wavelength range of 200~600 nm; the resulting absorption spectra are shown in Figure 1. From the figure, it can be seen that methamphetamine has its maximum absorption at a wavelength of 207 nm, but the terminal absorption at 207 nm is larger. However, considering the large absorption of the mobile phase at 207 nm, λ = 260 nm was selected as the UV detection wavelength for HPLC. Common HPLC detectors include ultraviolet-visible spectrophotometers, electrochemical detectors, fluorescence detectors, and MS [48]. HPLC-FL is more sensitive than HPLC-UV, and the limit of detection (pg on the column) is usually two orders of magnitude higher; however, the analysis time is shorter in HPLC-UV compared to HPLC-FL, and it is universally accessible for any laboratory HPLC-UV system, which is an important advantage of HPLC-UV.

#### 2.1.3. Selection of Chromatographic Column and Mobile Phase

The chromatographic column was a Spherisorb ODS2 C18 column (4.6 mm × 200 mm, 5 μm), which is widely used in HPLC as a packed column with a spherical silica packing material [49].

In LC analyses, water is generally used as the base solvent for the mobile phase, and methanol and acetonitrile are commonly used as organic mobile phase eluents because they can affect chromatographic separation and enhance the ionization of analytes [50]. Methanol was chosen in this experiment because using acetonitrile would result in a much lower signal, while a mobile phase composed of methanol/water would facilitate ionization and increase the observed signal (peak area). Adding a buffer solution to the mobile phase also plays a significant role in improving ionization efficiency and providing a better peak shape [51]. Phosphate buffers and triethylamine solutions are generally used when analyzing weakly basic materials. The methamphetamine hydrochloride in an adsorbed material will be in the form of a methamphetamine free base [8], and the use of a triethylamine buffer in the experiments enhances sensitivity, which is in agreement with a previous study [52]. Therefore, a 0.25% methanol/triethylamine aqueous solution (V:V = 20:80) was selected as the best mobile phase eluent.

The mobile phase was adjusted to an acidic pH with glacial acetic acid, pH = 3.1, and the pH of the mobile phase affects the dissociation of the analyzed components. Gray et al. [53] determined that alkaline mobile phases allow for better separation of isomers. However, alkaline mobile phases may shorten the life of HPLC columns, and column efficiency is difficult to maintain [54].

Under the above chromatographic conditions, the flow rate was 1.0 mL/min, the detection wavelength was 260 nm, the column temperature was 25 °C, and the injection volume was 20 μL. The results were that the number of theoretical plates for methamphetamine was ≥2000, and the degree of separation was 1.51. The efficiency of a chromatographic column is represented by the theoretical number of plates (N) [55], which was applied in chromatography by Martin and Synge in 1941 [56]. The resolution (R) is used to determine the separation of a substance in a chromatographic column. The larger the R value, the better the separation between adjacent substances. Generally, when R = 1.0, the separation is up to 98%; when R = 1.5, the separation is up to 99.7% [55].

#### 2.1.4. Optimization of Sample Pretreatment Methods

Pre-assay gauze was stored in a wide-mouth vial containing methamphetamine in an airtight chamber for 24 h. Similar to Morrison’s method, Morrison et al. [44] exposed fabric samples such as cotton and polyester (PE) to gaseous methamphetamine in a sealed chamber for 60 days and then extracted the fabric samples in liquid form. This study calculated the partition coefficient of methamphetamine between fabric and air (units: µg of methamphetamine per gram of substrate per ppb). The partition coefficients for cotton and PE at 30% RH were 18.3 ± 8.0 and 5.6 ± 3.5 µg/(g ppb), respectively.

### 2.2. Method Validation

The method developed in this study was validated by measuring selectivity, linearity, the limit of detection (LOD), the limit of quantitation (LOQ), precision, stability, and recovery.

#### 2.2.1. Selectivity

**Figure 2:**
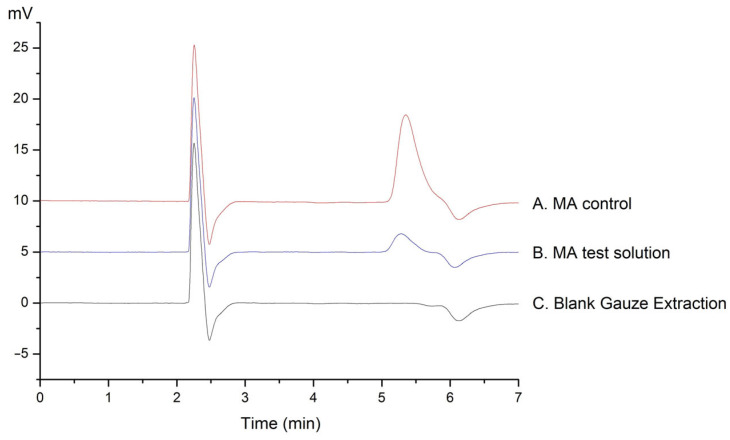
Chromatograms obtained from different carriers: (A) MA control, (B) MA test solution, and (C) blank gauze extraction.

The gauze blank extraction solution, the methamphetamine control solution, and the test solution were injected using our chromatographic method. The test solution did not show an interference peak during the retention time of the analyte. As can be seen from the chromatogram in Figure 2, the gauze blank extraction solution did not interfere with the determination of methamphetamine under these chromatographic conditions.

#### 2.2.2. LOD and LOQ

An appropriate amount of methamphetamine control solution was taken, gradually diluted with the mobile phase, and injected into the sample for determination according to the chromatographic conditions of this experiment; the results showed that the LOD was 1 μg/mL (S/N = 3), and the LOQ was 3 μg/mL (S/N = 10).

The LOD is used to examine whether a method has a sensitive detection ability. The LOQ reflects whether the analytical method has a sensitive quantitative detection capability. The LOD and LOQ are calculated based on the signal-to-noise ratio (S/N). The LOD and LOQ are often defined as S/N = 3 and S/N = 10 [57], respectively.

#### 2.2.3. Linearity and Range

Standard mixed solutions of the target detectives at series mass concentrations were prepared separately, and the drug concentration *X* (mg/mL) was linearly regressed against the peak area *Y* to obtain the regression equation of the calibration curve and the corresponding coefficient (*R*^2^). The regression equation was Y = 826.8X − 105.1, with a slope of 826.75 ± 34.92 and an intercept of 105.06 ± 9.17. Correlation coefficient (*R*^2^) = 0.999 9. The results showed that methamphetamine exhibited good linearity in the range of 4.2~83.2 μg/mL.

#### 2.2.4. Repeatability

Repeatability is the degree to which results remain unaffected by small changes in measurement conditions; it mainly examines the stability of the factors affecting a method to ensure that the method is accurate and reliable.

The peak area of methamphetamine was determined after the control solution of methamphetamine was repeatedly injected into the sample six consecutive times. The results showed that the peak area of methamphetamine was 50,589 ± 187, with a relative standard deviation (RSD) of 0.4%, n = 6. It indicated that the effect of the experimental results was not significant, the method was reproducible, and the reproducibility of the instrument was good.

#### 2.2.5. Precision

Precision refers to the degree of proximity (dispersion) among the results obtained from a series of tests on the same homogeneous sample taken several times under specified test conditions. HPLC results are mostly expressed in terms of RSD.

Six samples of methamphetamine gauze were measured under the chromatographic conditions in this experiment and then substituted into the regression equation to calculate the content. The resultant mean value of the methamphetamine content was 10.1 μg with an RSD of 1.5%, which indicated the good precision of the method. The RSD of precision was less than 15% within the permissible range [58].

#### 2.2.6. Stability

One sample of methamphetamine gauze was continuously detected under the chromatographic conditions of this experiment for 24 h. The RSD was 2.2% in terms of the peak area of methamphetamine, which indicated that the stability of the test material was good over a 24 h period. The precision RSD should be within ±15% [59].

#### 2.2.7. Recovery

Nine samples of blank gauze were added to an appropriate amount of control solution; determination was carried out under the chromatographic conditions of this experiment, and the spiked recoveries were calculated. The results are shown in Table 1. The average spiked recovery values of methamphetamine at high, medium, and low concentrations were 88.9 ± 0.7%, 85.5 ± 0.3%, and 84.3 ± 2.6%, respectively, with an RSD of 2.9%, which indicated that the method had good recovery values [60].

### 2.3. Application to Real Samples

Three samples of gauze which had adsorbed methamphetamine odor for 24 h were taken, the test solution was prepared according to 3.4, the chromatographic conditions were determined according to 3.3, and the content was calculated by the external standard method. The results showed that the gauze samples contained 7.8 ± 2.2 μg/sample of methamphetamine.

## 3. Materials and Methods

### 3.1. Chemicals and Reagents

Methamphetamine crystals (samples were collected from the Public Security Department of Liaoning Province, Jinzhou, China), methanol (Shandong Yuwang Group, Dezhou, China), triethylamine (Shanghai McLean Biochemistry Science and Technology Co., Ltd., Shanghai, China), glacial acetic acid (Sinopharm Chemical Reagent Co., Ltd., Shenyang, China), and a block of sterile gauze (Huaru Medical Company, 6 cm× 8 cm, Liaocheng, China) were obtained.

The methamphetamine-adsorbed gauze was prepared as follows: sterile tweezers were used to place gauze and methamphetamine crystals together in a sterile wide-mouth vial (750 mL) with a cap. The vial was stored indoors for 24 h at a temperature of 26 ± 1 °C and humidity of 50–60%.

### 3.2. Instrumentation

A high-performance liquid chromatograph (including an Autosampler L-2200, a High-Pressure Pump L-2130, and a UV Detector L-2420) was purchased from Hitachi (China) Co., Ltd., Shenyang, China; a UV-1700 Ultraviolet Spectrophotometer was purchased from Shimadzu, Japan; a Legend Micro17 Benchtop Centrifuge was purchased from Thermo Fisher Scientific, Waltham, MA, USA; and an SB-5200DTD Ultrasonic Cleaning Instrument was purchased from Ningbo Xinzhi Bio-technology Co., Ningbo, China.

### 3.3. Chromatographic Conditions

An appropriate amount of methamphetamine was taken, weighed precisely, dissolved, and diluted with methanol to make a solution with a concentration of 40 μg/mL with methanol as the reference solution and then scanned in the wavelength range of 200~600 nm. The UV detection wavelength for HPLC was selected.

The separation was performed using a Spherisorb ODS2 C18 column (4.6 mm × 200 mm, 5 μm) with the mobile phase consisting of a 0.25% methanol/triethylamine aqueous solution (V:V = 20:80), and the pH was adjusted to 3.1 using glacial acetic acid at a flow rate of 1.0 mL/min. The column temperature was 25 °C. A sample volume of 20 μL was injected, and the detection of the analytes was performed at a wavelength of 260 nm.

### 3.4. Preparation of Solutions

#### 3.4.1. Preparation of Control Solution

First, 10.2 mg of methamphetamine control was weighed and placed into a 50 mL volumetric flask; methanol was added to dissolve it, and it was diluted according to the scale to obtain a control stock solution with a concentration of 204 μg/mL. Then, 2.5 mL of control stock solution was measured, placed into a 10 mL volumetric flask, and diluted with water; thus, a control solution with a concentration of 51 μg/mL was obtained.

#### 3.4.2. Preparation of Test Solution

First, 4 cm^2^ of gauze with adsorbed methamphetamine was taken and placed into a 1.5 mL EP tube; 1 mL of methanol was added, the sample was sonicated for 10 min and then filtered using a 0.45 μm microporous filter membrane. The filtrate was taken as the test solution.

### 3.5. Method Validation

#### 3.5.1. Selectivity Test

The gauze blank extraction solution, the methamphetamine control solution, and the test solution were injected separately according to the chromatographic conditions described in Section 3.3 to check possible interferences for the retention time of the analytes.

#### 3.5.2. LOD and LOQ Tests

An appropriate amount of the methamphetamine control solution was taken, diluted gradually with the mobile phase, and injected into the sample for determination according to the chromatographic conditions described in Section 3.3. The LOD and LOQ were estimated for signal-to-noise ratios (S/Ns) of 3 and 10.

#### 3.5.3. Linearity Test

First, 4.0, 2.0, 2.5, 0.8, 0.5, 0.5, and 0.5 mL samples of the methamphetamine control stock solution were placed in 10, 10, 25, 10, 10, 10, and 25 mL volumetric flasks, respectively, diluted with methanol, and fixed to the scale, shaking well. Standard solutions of methamphetamine were prepared at concentrations of 83.2, 41.6, 20.8, 16.6, 10.4, and 4.2 mg/mL. The sample was injected in a volume of 20 μL, and the drug concentration X (mg/mL) was linearly regressed against the peak area Y. The regression equation and correlation coefficient were calculated.

#### 3.5.4. Repeatability Test

A volume of 20 μL of the methamphetamine control solution was precisely drawn, and the injection was repeated 6 times continuously under the chromatographic conditions described in Section 3.3 to determine the peak area of methamphetamine.

#### 3.5.5. Precision Test

Six samples of gauze with adsorbed methamphetamine were taken, and the test solution was prepared according to the preparation method described in Section 3.4. Measurements were taken according to the chromatographic conditions described in Section 3.3, and the content was calculated by substituting the value into the regression equation.

#### 3.5.6. Stability Test

One sample of gauze with adsorbed methamphetamine was taken; the test solution was prepared and left for 0, 4, 8, 12, and 24 h at room temperature. Then, the content was determined according to the chromatographic conditions described in Section 3.3.

#### 3.5.7. Recovery Test

Nine samples of blank gauze in EP tubes were taken, and an appropriate amount of control solution was added to each tube, respectively. The test solution was prepared according to the preparation method described in Section 3.4, and measurements were taken according to the chromatographic conditions described in Section 3.3; then, the spiked recovery was calculated.

## 4. Conclusions

In this study, we developed a relatively simple, rapid, and accurate HPLC method for the qualitative and quantitative analysis of methamphetamine in an adsorbent material (gauze). The main advantage of our method is the simplicity of sample preparation and instrumentation, which are suitable for routine monitoring and analysis in most laboratories. The applicability of the method for the detection of methamphetamine in a routine adsorbent material (gauze) was demonstrated by analyzing real methamphetamine samples. These techniques can be used to detect the content of target odors in adsorbent materials during animal training to enhance drug recognition and control by drug detection animals.

## Figures and Tables

**Table 1:** Recovery test results.

No.	Volume of the Control (μg)	Total Actual Test Volume (μg)	Recovery (%)	Average Recovery (%)	SD (%)	RSD (%)
1	41.6	36.7	88.3	88.9	0.7	2.9
2	41.6	37.3	89.6			
3	41.6	37.0	89.0			
4	20.8	17.8	85.5	85.5	0.3	
5	20.8	17.7	85.2			
6	20.8	17.8	85.7			
7	8.3	6.9	82.5	84.3	2.6	
8	8.3	7.3	87.3			
9	8.3	6.9	83.2			

## Data Availability

Data are contained within the article.
